# TRIM41-Mediated Ubiquitination of Nucleoprotein Limits Vesicular Stomatitis Virus Infection

**DOI:** 10.3390/v12020131

**Published:** 2020-01-22

**Authors:** Girish Patil, Lingling Xu, Yakun Wu, Kun Song, Wenzhuo Hao, Fang Hua, Lingyan Wang, Shitao Li

**Affiliations:** 1 Department of Physiological Sciences, Center for Veterinary Health Sciences, Oklahoma State University, 156 McElroy Hall, Stillwater, OK 74078, USA; girisnp@ostatemail.okstate.edu (G.P.); Lingling.Xu@uthct.edu (L.X.); 2 Department of Microbiology and Immunology, Tulane University, New Orleans, LA 70112, USA; ywu36@tulane.edu (Y.W.); ksong@ostatemail.okstate.edu (K.S.); wenzhuo.hao@okstate.edu (W.H.); fhua@tulane.edu (F.H.); lwang32@tulane.edu (L.W.)

**Keywords:** TRIM, ubiquitination, host defense, intrinsic immunity, proteasomal degradation

## Abstract

Vesicular stomatitis virus (VSV) is a zoonotic, negative-stranded RNA virus of the family Rhabdoviridae. The nucleoprotein (N) of VSV protects the viral genomic RNA and plays an essential role in viral transcription and replication, which makes the nucleoprotein an ideal target of host defense. However, whether and how host innate/intrinsic immunity limits VSV infection by targeting the N protein are unknown. In this study, we found that the N protein of VSV (VSV-N) interacted with a ubiquitin E3 ligase, tripartite motif protein 41 (TRIM41). Overexpression of TRIM41 inhibited VSV infection. Conversely, the depletion of TRIM41 increased host susceptibility to VSV. Furthermore, the E3 ligase defective mutant of TRIM41 failed to limit VSV infection, suggesting the requirement of the E3 ligase activity of TRIM41 in viral restriction. Indeed, TRIM41 ubiquitinated VSV-N in cells and in vitro. TRIM41-mediated ubiquitination leads to the degradation of VSV-N through proteasome, thereby limiting VSV infection. Taken together, our study identifies TRIM41 as a new intrinsic immune factor against VSV by targeting the viral nucleoprotein for ubiquitination and subsequent protein degradation.

## 1. Introduction

Vesicular stomatitis virus (VSV) belongs to the Rhabdoviridae that encapsidates a single-stranded RNA genome encoding five major proteins [1]. VSV is a zoonotic virus with epidemic potential in horse, cattle, and swine, which causes significant economic burden to the livestock industry [2]. To infect host cells, VSV binds to the cell receptors through its glycoprotein (G), which triggers endocytosis followed by uncoating of the helical nucleocapsid (NC) in the cytoplasm. The nucleoprotein (N) encapsidates the viral RNA to form the NC, which is the template for full-length NC replication and for transcription of subgenomic mRNAs [3,4]. The NC is tightly shielded by N protein and only opens transiently for RNA synthesis by the L polymerase and phosphoprotein (P) [5,6]. Since the N protein is critical for VSV capsid and viral replication, it is an ideal target for the host immune system. However, whether and how the N protein is targeted by host innate or intrinsic immunity are unknown. Filling this knowledge gap will not only deepen our understanding of host defense to VSV but also pave an avenue for the development of therapeutics for VSV infectious disease.

The tripartite motif (TRIM) proteins are a group of ubiquitin E3 ligases that play critical roles in innate immune signaling as well as intrinsic immunity. The ubiquitin E3 ligase activity is conferred by the conserved N-terminal motif comprising three domains, Really Interesting New Gene (RING), B box, and coiled coil. TRIM proteins have variable C-terminal domains that determine the interaction between the target proteins and TRIM proteins. It is well known that TRIM proteins mediate host innate immune activation and promote the induction of antiviral cytokines and chemokines, such as type I interferon (IFN). For instance, TRIM25 ubiquitinates the cytosolic RNA sensor, retinoic acid-inducible gene I (RIG-I), which is critical for subsequent RIG-I oligomerization and activation [7,8]. TRIM5 interacts with the human immunodeficiency virus (HIV) capsid and activates NF-κB signaling [9]. Recent studies found that TRIM proteins are also intrinsic immune factors, which limit virus infection by directly targeting viral proteins [10]. For example, TRIM32 ubiquitinates the polymerase basic 1 (PB1) of influenza A virus, which leads to PB1 proteasomal degradation, thereby inhibiting viral infection [11].

Several TRIM proteins, including TRIM41, participate in both innate immunity and intrinsic immunity. First, TRIM41 regulates nucleotide binding oligomerization domain containing 2 (NOD2) and cyclic GMP-AMP synthase (cGAS) innate immune signaling pathways. TRIM41 interacts with NOD2, but the underlying mechanism by which TRIM41 regulates NOD2 signaling is not clear [12]. TRIM41 also mediates monoubiquitination of the cytosolic DNA sensor cGAS, thereby promoting type I IFN production [13]. Secondly, TRIM41 is an intrinsic immunity factor that inhibits viral infection. Our recent study discovered that TRIM41 restricts influenza by polyubiquitination and degradation of viral nucleoprotein [14]. Moreover, a recent screening of TRIM proteins found that TRIM41 and other seven TRIM proteins inhibited the hepatitis B virus (HBV) transcription [15]. However, whether TRIM41 inhibits other viruses, such as VSV, is unknown.

In this study, we demonstrate that TRIM41 inhibited VSV infection by both gain- and loss-of-function studies. Co-immunoprecipitation found that TRIM41 interacted with the N protein of VSV (VSV-N). Furthermore, TRIM41 ubiquitinated VSV-N in cells and in vitro, which led to proteasomal degradation of VSV-N, thus limiting VSV replication. Overall, our study establishes TRIM41 as a new intrinsic immune host factor against VSV.

## 2. Materials and Methods

### 2.1. Cells and Viruses

HEK293 cells (ATCC, # CRL-1573) and Vero cells (ATCC, # CCL-81) were maintained in Dulbecco’s Modified Eagle Medium (Life Technologies, Carlsbad, CA, USA) containing Penicillin-Streptomycin (Life Technologies) and 10% fetal bovine serum (Life Technologies). A549 cells (ATCC, # CCL-185) were cultured in RPMI Medium 1640 (Life Technologies) plus 10% fetal bovine serum and 1× MEM Non-Essential Amino Acids Solution (Life Technologies).

VSV Indiana strain was purchased from ATCC (VR-1238). The VSV carrying a luciferase gene (VSV-Luc) and the VSV expressing a GFP gene (VSV-GFP) were a kind gift from Dr. Sean Whelan (Harvard Medical School, MA, USA). Viral titration was determined in Vero cells. Briefly, Vero cells were infected with a serial diluted VSV. After 45 min, the medium was removed and replaced by the DMEM plus 10% FBS and 1% agarose. After 48 h, cells were fixed using the methanol–acetic acid (3:1) fixative and stained using a Coomassie blue solution.

### 2.2. Constructs and Reagents

TRIM41-HA was a generous gift from Dr. Adolfo Garcia-Sastre (Mount Sinai School of Medicine, NY, USA). TRIM41 was cloned into pCMV-3Tag-8 (Stratagene, San Diego, CA, USA) to generate TRIM41-FLAG. Deletion mutants of TRIM41-FLAG were constructed using a Q5^®^ Site-Directed Mutagenesis Kit (New England Biolabs, Ipswich, MA, USA). VSV-N was cloned into pCMV-3Tag-8 (Stratagene) and pCMV-3Tag-8-HA to generate FLAG-tagged and HA-tagged VSV-N, respectively.

Anti-β-actin (Abcam, # ab8227, Cambridge, UK), anti-FLAG (Sigma, # F3165, St. Louis, MO, USA), anti-ubiquitin (Santa Cruz Biotechnology, # sc-8017, Dallas, TX, USA), anti-TRIM41 (Aviva Systems Biology, # ARP34763_P050, San Diego, CA, USA), anti-HA epitope (Cell Signaling Technology, # 3724, Danvers, MA, USA), anti-VSV (Imanis Life Sciences, # REA005, Rochester, MN, USA), and anti-VSV-N (KeraFAST, # EB0009, Boston, MA, USA). Goat anti-Mouse IgG-HRP (Santa Cruz Biotechnology, # sc-2055), Goat anti-Rabbit IgG-HRP (Santa Cruz Biotechnology, # sc-2030), Alexa Fluor 594 Goat Anti-Mouse IgG (H+L) (Life Technologies, # A11005), and Alexa Fluor 488 Goat Anti-Rabbit IgG (H+L) (Life Technologies, # A11034).

### 2.3. Sample Preparation, Western Blotting, and Immunoprecipitation

Approximately 1 × 10^6^ cells were lysed in 500 µL of tandem affinity purification (TAP) lysis buffer [50 mM Tris-HCl (pH 7.5), 10 mM MgCl_2_, 100 mM NaCl, 0.5% Nonidet P40, 10% glycerol, Complete EDTA-free protease inhibitor cocktail tablets (Roche, Basel, Switzerland)] for 30 min on ice. The lysates were then centrifuged for 30 min at 15,000× *g*. Supernatants were collected and mixed with 1× Lane Marker Reducing Sample Buffer (Thermo Fisher Scientific, Waltham, MA, USA). Western blotting and immunoprecipitation were performed as described in a previous study [16].

### 2.4. Immunofluorescence Assay

Cells were cultured in the Lab-Tek II CC2 Chamber Slide System 4-well (Thermo Fisher Scientific). After the indicated treatment, the cells were fixed and permeabilized in cold methanol for 10 min at −20 °C. Then, the slides were washed with 1× DPBS for 10 min and blocked with Odyssey Blocking Buffer (LI-COR Biosciences, Lincoln, NE, USA) for 1 h. The slides were incubated in Odyssey Blocking Buffer with 1:100 diluted primary antibodies at 4 °C for 12 h. Images were captured and analyzed using an iRiS^TM^ Digital Cell Imaging System (Logos Biosystems, Annandale, VA, USA).

### 2.5. Plasmid Transfection

Transfections using Lipofectamine 2000 or Lipofectamine 3000 transfection reagent (Life Technologies) were performed according to the manufacturer’s protocol. For co-IP experiments, a total of 2.5 µg of plasmids was transfected into approximately 1.2 × 10^6^ cells. For other experiments, a total of 0.5 µg of plasmids was transfected into approximately 0.2 × 10^6^ cells.

### 2.6. RNA Interference

RNAi target sequences (sense strand): TRIM41 siRNA #2: AAGGCGTGCTGTGGAAATAAA; TRIM41 siRNA #3: TTCAATAGGTGTGAAGAGGTA. siGENOME Non-Targeting Control siRNA (Dharmacon, # D-001210-02-05) was used as the control siRNA. Five pmol of siRNA duplexes were transfected into A549 cells using Lipofectamine RNAiMAX Transfection Reagent (Life Technologies) according to the manufacturer’s protocol.

### 2.7. CRISPR/Cas9

The single guide RNA (sgRNA) sequence targeting human TRIM41 is GTAGTCTTCATCCCGCATGG. The sgRNA was cloned into LentiCRISPR v2 [17] (Addgene, Cambridge, MA, USA). 0.5 µg of the lentiviral construct was transfected into HEK293 cells using Lipofectamine 2000. Cells were selected with 10 µg/mL puromycin for 14 days. Single clones were expanded for knockout confirmation by Western blotting and DNA sequencing.

### 2.8. In Vitro Ubiquitination

In vitro ubiquitination assay was performed according to the manufacturer’s manual (Boston Biochem, Cambridge, MA, USA). Ubiquitin, E1, UBCH5A (Boston Biochem), VSV-N-FLAG, and TRIM41-HA or Del-RING-HA bound to the anti-HA resin (Sigma) were incubated at 30 °C in the ubiquitin assay reaction buffer (Boston Biochem) for 2 h. The anti-HA resin was washed with 1 M urea for 15 min to exclude potential binding of unanchored polyubiquitin. Then the resin was incubated with 45 µL of 0.5 mg/mL HA peptide to elute VSV-N protein. The eluates were subsequently analyzed by SDS-PAGE, followed by Western blotting.

### 2.9. Statistical Analysis

The sample size was sufficient for data analysis using paired two-tailed Student’s *t*-test. For all statistical analyses, differences were considered to be statistically significant at values of *p* < 0.05.

## 3. Results

### 3.1. TRIM41 Restricts VSV Infection

**Figure 1:**
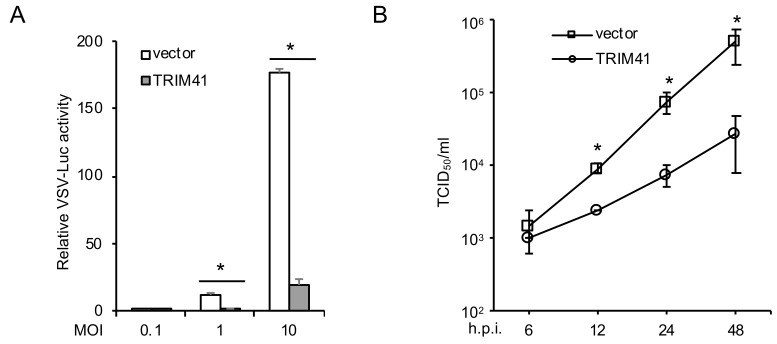
Ectopic expression of TRIM41 inhibits VSV infection. (**A**) HEK293 cells transfected with FLAG-tagged TRIM41 (TRIM41-FLAG) or pCMV-3Tag-8 vector were infected with the designated multiplicity of infections (MOIs) of VSV-Luc for 12 h. Relative VSV activities were determined by the luciferase activities that were normalized to the control. Data represent means ± s.d. of three independent experiments. The *p* value was calculated (two-tailed Student’s *t*-test) by comparison with the vector control. An asterisk indicates *p* < 0.05. (**B**) HEK293 cells were transfected with pCMV-3Tag-8 vector or TRIM41-FLAG. After 24 h, cells were infected with 0.001 MOI of VSV. After the designated hour post-infection (h.p.i.), virus titers were determined by TCID_50_ in Vero cells. All experiments were biologically repeated three times. The *p* value was calculated (two-tailed Student’s *t*-test) by comparison with the vector control. An asterisk indicates *p* < 0.05.

To examine the effect of TRIM41 on VSV infection, we first transfected FLAG-tagged TRIM41 into HEK293 cells. After 48 h, cells were infected with a VSV reporter virus carrying a luciferase gene in the viral genome (VSV-Luc). As shown in Figure 1A, the ectopic expression of TRIM41 inhibited VSV replication activity. To corroborate this finding, we performed TCID_50_ assay to determine the effect of TRIM41 overexpression on the production of infectious VSV particles. Overexpression of TRIM41 reduced VSV viral titers significantly at the time course of 6 h to 48 h. (Figure 1B). Taken together, these data suggest that TRIM41 is an anti-VSV host factor.

### 3.2. TRIM41 Deficiency Increases Host Susceptibility to VSV

**Figure 2:**
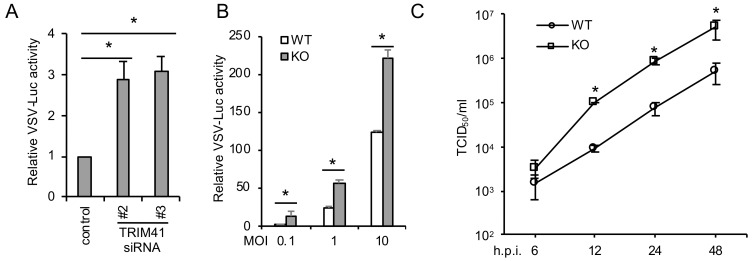
Depletion of TRIM41 increases host susceptibility to VSV infection. (**A**) A549 cells were transfected with 5 pmol of the control siRNA or the indicated siRNA duplex against TRIM41. After 48 h, the cells were infected at an MOI of 0.1 with VSV-Luc for 12 h. Relative VSV activities were determined by the luciferase activities that were normalized to the control. All experiments were biologically repeated three times. Data represent means ± standard deviations of three independent experiments. The *p* value was calculated (two-tailed Student’s *t*-test) by comparison of results with those of the siRNA control in each cell group. An asterisk indicates *p* < 0.05. (**B**) Wild type (WT) and TRIM41 knockout (KO) HEK293 cells were infected with the indicated MOIs of VSV-Luc for 12 h. Relative VSV activities were determined by the luciferase activities that were normalized to the control. All experiments were biologically repeated three times. The *p* value was calculated (two-tailed Student’s *t*-test) by comparison with the wild type. An asterisk indicates *p* < 0.05. (**C**) Wild type and TRIM41 knockout cells were infected with 0.001 MOI of VSV. After the designated hour post-infection, virus titers were determined by TCID_50_ in Vero cells. All experiments were biologically repeated three times. The *p* value was calculated (two-tailed Student’s *t*-test) by comparison with the wild type. An asterisk indicates *p* < 0.05.

To corroborate the gain-of-function of TRIM41, we further examined the effect of TRIM41 depletion on VSV infection. We first depleted TRIM41 using small interfering RNA (siRNA). Two validated siRNA duplexes against TRIM41 [14] were individually transfected into A549 lung epithelial cells. After 48 h, cells were infected with VSV-Luc for 12 h. Knockdown of TRIM41 increased VSV infection activity in A549 cells (Figure 2A). Secondly, wild type and the TRIM41 knockout HEK293 cells used in our previous study [14] were infected with different doses of VSV-Luc for 12 h. Reporter assays demonstrated the increased viral infection in TRIM41 knockout cells (Figure 2B). Lastly, viral titers were determined by TCID50 assay in TRIM41 wild type vs. knockout cells. VSV viral titers increased about 10-fold in knockout cells compared to wild type cells (Figure 2C), suggesting depletion of TRIM41 impairs host defense to VSV infection.

### 3.3. TRIM41 Interacts with the Nucleoprotein of VSV

**Figure 3:**
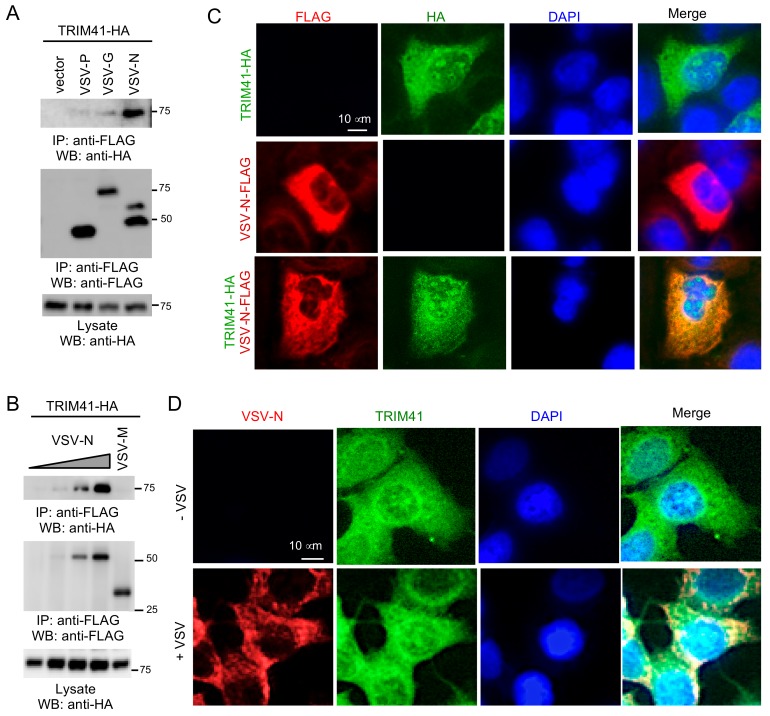
TRIM41 co-localizes and interacts with the N protein of VSV. (**A**) HA-tagged TRIM41 (TRIM41-HA) was co-transfected with FLAG-tagged phosphoprotein (P), glycoprotein (G), or nucleoprotein (N) of VSV into HEK293 cells. Cells were treated with 1 µM proteasome inhibitor MG132 to prevent VSV protein from degradation. Cell lysates were immunoprecipitated with the anti-FLAG antibody and then blotted as indicated. (**B**) TRIM41-HA was co-transfected with FLAG-tagged matrix protein (M) or different doses of nucleoprotein (N) of VSV into HEK293 cells. Cells were treated with 1 µM proteasome inhibitor MG132 to prevent VSV protein from degradation. Cell lysates were immunoprecipitated with the anti-FLAG antibody and then blotted as indicated. (**C**) A549 cells were transfected with TRIM41-HA and FLAG-tagged VSV-N (VSV-N-FLAG). Cells were treated with 1 µM MG132 to prevent VSV protein from degradation. Red: FLAG; Green: HA; Blue: DAPI, a nuclear stain. (**D**) A549 cells were mock-infected or infected with VSV. Cells were treated with 1 µM MG132 to prevent VSV-N from degradation. After 24 h, cells were fixed with cold methanol and incubated with anti-VSV-N and anti-TRIM41 antibodies. Red: VSV-N; Green: TRIM41; Blue: DAPI, a nuclear stain.

We previously reported that TRIM41 interacted with influenza viral protein to limit viral infection [14]. Therefore, it is plausible that TRIM41 also interacts with VSV protein(s) to inhibit viral infection. In this regard, FLAG-tagged G, P, M, or N of VSV was co-transfected with HA-tagged TRIM41 into HEK293 cells. Co-immunoprecipitation (co-IP) revealed the specific interaction between TRIM41 and the N protein of VSV (VSV-N) (Figure 3A,B). Next, we examined the subcellular localization of TRIM41 and VSV-N by immunofluorescence assay. FLAG-tagged VSV-N and HA-tagged TRIM41 were transfected alone or together into A549 cells. The immunofluorescence assays showed that TRIM41 expressed in the cytoplasm and the nucleus, while VSV-N exclusively expressed in the cytoplasm (Figure 3C). TRIM41 co-localized with VSV-N in the cytoplasm (Figure 3C). We further examined the co-localization of endogenous TRIM41 and VSV-N during viral infection. A549 cells were cultured in a chamber slide and infected with 1 MOI of VSV for 24 h. Consistent with the ectopic expressing TRIM41, the endogenous TRIM41 was expressed in the cytoplasm and the nucleus (Figure 3D). VSV-N only expressed in the cytoplasm and co-localized with TRIM41 in the cytoplasm (Figure 3D). These data suggest that TRIM41 interacts and co-localizes with VSV-N.

### 3.4. E3 Ligase Activity Is Required for TRIM41 Antiviral Function

**Figure 4:**
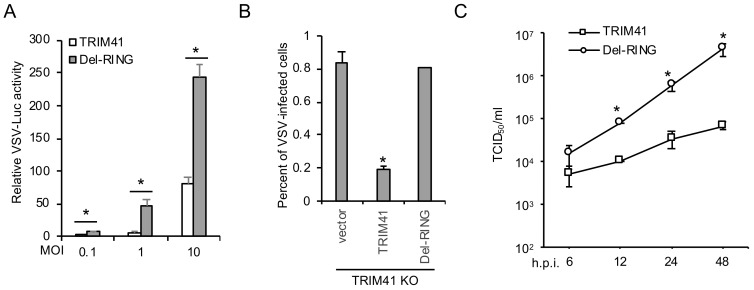
E3 ligase activity is required for TRIM41 anti-VSV function. (**A**) HEK293 cells were transfected with FLAG-tagged TRIM41 or the RING deletion mutant (Del-RING). After 24 h, cells were infected with designated MOIs of VSV for 12 h. Relative VSV activities were determined by the luciferase activities that were normalized to the control. Data represent means ± s.d. of three independent experiments. The *P* value was calculated by two-tailed Student’s *t*-test. An asterisk indicates *p* < 0.05. (**B**) TRIM41 knockout HEK293 cells were transfected with pCMV-3Tag-8 vector, TRIM41-FLAG, or the Del-RING for 24 h, then infected with 0.1 MOI of VSV-GFP for 12 h. The percentage of GFP-positive cells was adopted to determine infection activity. An asterisk indicates *p* < 0.05. (**C**) TRIM41 HEK293 knockout cells were transfected with wild type TRIM41 or the Del-RING mutant for 24 h, followed by infection of 0.001 MOI of VSV. After the designated hour post-infection, virus titers were determined by TCID_50_ in Vero cells. All experiments were biologically repeated three times. The *p* value was calculated (two-tailed Student’s *t*-test) by comparison with the wild type. An asterisk indicates *p* < 0.05.

TRIM proteins are ubiquitin E3 ligases; thus, we examined whether the enzymatic activity of TRIM41 is required for its anti-VSV function. Since the RING domain confers E3 ubiquitin ligase activity to TRIM proteins, we first examined the effect of RING deletion mutant (Del-RING, aa 34–aa 76 deleted) on VSV infection. TRIM41 or the Del-RING was transfected into HEK293 cells, followed by infection with different MOIs of VSV-Luc for 16 h. Luciferase reporter assays showed that Del-RING failed to inhibit VSV infection (Figure 4A). We further reconstituted full-length TRIM41 or the Del-RING in TRIM41 knockout cells by transfection. After 48 h, cells were infected with the VSV carrying a GFP (VSV-GFP). As shown in Figure 4B, full-length TRIM41, but not the Del-RING, restored antiviral activity against VSV in the knockout cells. Lastly, we examined viral titers in TRIM41 knockout cells reconstituted with full-length TRIM41 vs. the Del-RING. In line with the reporter assays, the Del-RING failed to suppress VSV infection evidenced by TCID_50_ assay (Figure 4C). Taken together, the ubiquitin E3 ligase activity is indispensable for the antiviral function of TRIM41.

### 3.5. TRIM41 Mediates the Ubiquitination and Degradation of VSV-N

**Figure 5:**
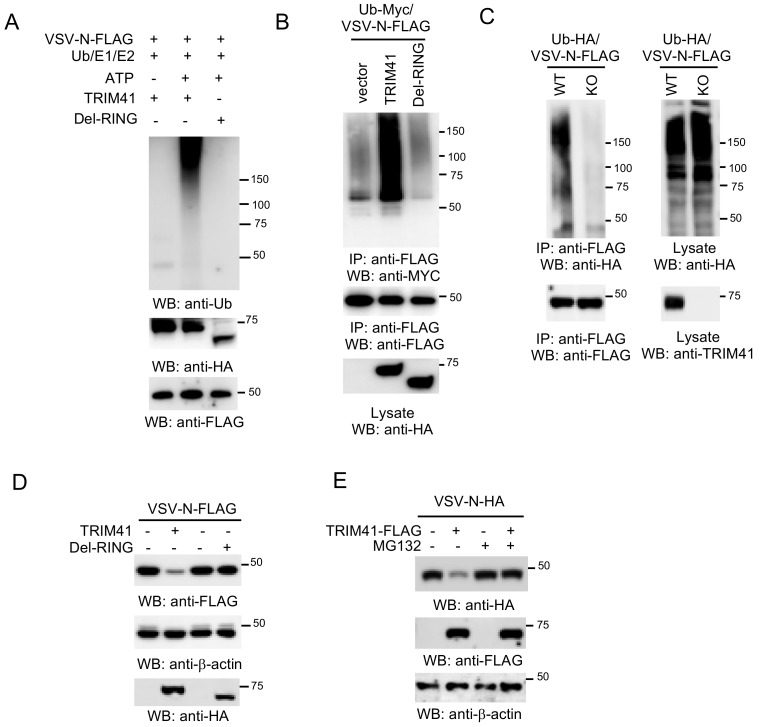
TRIM41 ubiquitinates and degrades the N protein of VSV. (**A**) In vitro ubiquitination of VSV-N by TRIM41. FLAG-tagged VSV-N, HA-tagged TRIM41, and the Del-RING mutant, plus E1, E2 (UBCH5A), ATP, and ubiquitin were added as indicated and incubated at 30 °C for 2 h. Samples were blotted with the indicated antibodies. (**B**) FLAG-tagged VSV-N was co-transfected with vector, TRIM41-HA, or the HA-tagged Del-RING mutant together with Myc-tagged ubiquitin (Ub-Myc) into HEK293 cells. Cells were treated with 1 µM proteasome inhibitor MG132 to prevent VSV-N from degradation. Cell lysates were immunoprecipitated with anti-FLAG antibody and blotted with the indicated antibodies. (**C**) Wild type and TRIM41 knockout HEK293 cells were transfected with FLAG-tagged VSV-N and HA-tagged ubiquitin (Ub-HA). Cell lysates were immunoprecipitated with anti-FLAG antibody and blotted with the indicated antibodies. (**D**) FLAG-tagged VSV-N was co-transfected with HA-tagged TRIM41 or the Del-RING mutant into HEK293 cells. Cell lysates were blotted as indicated. (**E**) HA-tagged VSV-N (VSV-N-HA) was co-transfected with vector or TRIM41-FLAG into HEK293 cells. After 24 h, cells were treated with DMSO or 1 µM MG132 for 12 h. Cell lysates were then blotted with the indicated antibodies.

The requirement of E3 ubiquitin ligase activity suggests that TRIM41 might inhibit VSV infection through the ubiquitination of VSV-N. To examine whether TRIM41 can conjugate ubiquitin molecules onto VSV-N, we first performed an in vitro ubiquitination assay. As shown in Figure 5A, TRIM41, but not the Del-RING, conjugated the ubiquitin molecules onto VSV-N. Consistently, overexpression of wild type TRIM41, but not the Del-RING mutant, induced the ubiquitination of VSV-N in HEK293 cells (Figure 5B). Next, we examined VSV-N ubiquitination in TRIM41 knockout cells. VSV-N was transfected with HA-tagged ubiquitin into wild type and TRIM41 knockout HEK293 cells. Knockout of TRIM41 dramatically reduced the levels of polyubiquitin chain conjugated onto VSV-N (Figure 5C). These experiments suggest that TRIM41 mediates polyubiquitination of VSV-N. Polyubiquitination often leads to proteasomal degradation of the targeted protein. Thus, we further examined whether TRIM41 mediates VSV-N protein degradation. VSV-N was co-transfected with TRIM41 or the Del-RING into HEK293 cells. Overexpression of full-length TRIM41 reduced the VSV-N expression; however, the Del-RING had a marginal effect on the expression of VSV-N protein (Figure 5D). Furthermore, we treated cells with the protease inhibitor MG132 to determine whether TRIM41-mediated protein degradation of VSV-N is proteasome-dependent. HEK293 cells were transfected with VSV-N along with TRIM41 or vector. After 48 h, cells were treated with the proteasome inhibitor MG132 for 6 h. Western blot analysis found that MG132 treatment blocked TRIM41-mediated VSV-N degradation (Figure 5E). In all, the combined data indicate that TRIM41 mediates the ubiquitination of VSV-N, which leads to VSV-N proteasomal degradation, thereby inhibiting VSV infection.

### 3.6. TRIM41 Restricts Incoming VSV Nucleocapsids

**Figure 6:**
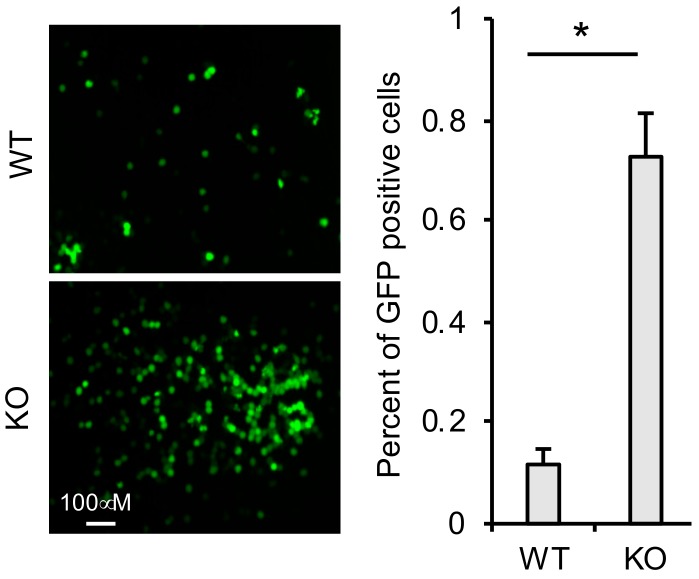
TRIM41 restricts incoming VSV nucleocapsids. TRIM41 wild type and knockout cells were infected with 1 MOI of VSV-GFP for 1 h. Then, cells were washed with PBS for two times to remove the unattached viruses. The neutralizing anti-VSV antibodies were added into the medium to prevent new infection. After 6 h, GFP positive cells were counted. The percentage of GFP-positive cells is summarized in the right graph. An asterisk indicates *p* < 0.05.

To determine whether TRIM41 can target the VSV-N of incoming VSV in the cells, we first infected TRIM41 wild type and knockout cells with the VSV-GFP. After 1 h, we washed away VSV in the medium and added the anti-VSV antibody to neutralize any newly released VSV virion. Therefore, the cells will only have a single cycle infection. As shown in Figure 6, TRIM41 knockout cells were more susceptible to the single cycle infection than wild type cells, suggesting that TRIM41 limits VSV infection by targeting the VSV-N of incoming virus.

## 4. Discussion

Innate immunity and intrinsic immunity are the frontlines of host defense to the invading viruses. Innate immunity comprises various signaling cascades leading to the expression of type I IFN and many other antiviral genes. These innate immune signaling pathways are triggered by the products and the by-products of microbes, which are usually foreign to the host. The Toll-like receptor 7 (TLR7) and RIG-I are the sensors that recognize the 5’-ppp-RNA of VSV in the endosome and the cytoplasm, respectively [18,19]. Upon the engagement with viral RNA, TLR7 and RIG-I activate signaling cascades, which lead to the induction of type I IFN expression [20,21,22,23,24]. Unlike innate immunity factors, the intrinsic immunity factors are a group of constitutively expressed host factors. The “pre-existed” expression of these host factors guarantees a more rapid response and a direct inhibition of viral infection [10,25]. For example, the cytidine deaminase, apolipoprotein editing complex 3 G (APOBEC3G), introduces transversion mutations into the HIV genome, thereby impeding HIV infection [26]. It is well established for how innate immunity responds to VSV infection; however, the intrinsic immune response to VSV is still elusive. Here, we report that TRIM41 interacts with the N protein of VSV and subsequently targets it for ubiquitination and proteasomal degradation. Our previous study showed that TRIM41 is constitutively expressed [14]. Thus, TRIM41 is a new intrinsic immune factor to VSV.

TRIM41 belongs to the TRIM family that consists of more than 70 members. Recently, many TRIM proteins are identified as intrinsic immune factors that curb viral infection through directly targeting viral proteins. For instance, TRIM32 restricts influenza A virus by targeting PB1 protein for ubiquitination and degradation [11]. TRIM22 and TRIM14 restrict hepatitis C virus by targeting NS5A for ubiquitination and degradation [27,28]. TRIM52 interacts with the nonstructural protein 2A of Japanese encephalitis virus and mediates NS2A ubiquitination and protein degradation [29]. Our study showed that TRIM41 inhibited VSV infection by mediating proteasomal degradation of the N protein. It should be noted that another TRIM protein, PML (also known as TRIM19), has been implied a role in VSV infection. Overexpression of PML in CHO cells increased the resistance to infection of VSV [30]. Furthermore, PML deficiency increases mice susceptibility to VSV infection [31]. A recent mechanistic study showed that PML enhances IFN synthesis by regulating the cellular distribution of Pin1 (peptidyl-prolyl cis/trans isomerase). The interaction of PML with endogenous Pin1 prevents the degradation of activated IRF3, thereby potentiating IRF3-dependent production of IFN [32].

Our previous study demonstrates that TRIM41 directly restricts influenza A virus by ubiquitination and degradation of nucleoprotein [14]. TRIM41 also inhibits HBV activity, which is dependent on the ubiquitin E3 ligase activity and the C-terminal domain of TRIM41 [15]. Thus, TRIM41 has emerged as a broad-spectrum antiviral factor. Like TRIM41, several other TRIM proteins are reported to have a broad-spectrum antiviral activity. PML also inhibits herpes virus and adeno-associated virus [33]. TRIM56 restricts influenza B virus, HIV, yellow fever virus, and dengue virus by reducing the viral RNA levels through its C-terminal tail [34,35]. TRIM22 restricts encephalomyocarditis virus, influenza A virus, hepatitis B virus, and hepatitis C virus [27,36,37,38]. However, the underlying mechanism for the broad-spectrum antiviral activity of TRIM proteins is unknown. To determine the broad-spectrum antiviral activity, it is important to identify the common mechanism. Future studies will determine whether the interacting viral proteins share a common motif for TRIM41 interaction.

In conclusion, we demonstrate that TRIM41 targets the N protein to inhibit VSV infection. The N protein covers the entire VSV genomic RNA and plays an important role in viral transcription and replication, which makes the N protein an ideal drug target. Thus, our study provides a foundation for future development of antiviral therapeutics.
